# 

**Published:** 1916-04-15

**Authors:** 


### CONTENTS.

PAGE

**The Man and the Moment** ... ... ... ... 41

**The New Taxes** ... ... ... ... ... ... ... 42

**Hospital and Institutional News** ... ... ... 43

The Horrors of Wittenberg—Two Medical Octogenarians—A Tribute to Hospital Treatment—Team Work in Plastic Surgery—An Assistant Medical Officers' Peerage—A New Medical Journal—The Late Colonel Barker—"Tom Bates, Surgeon"—The Uniform System at Newbury—Leicester Royal Infirmary—Bristol's Red Cross Record—A Striking Testimonial—V.A.D. Nurses and the Worcester Infirmary—The Hospital Standard at Worcester—Carnarvon Infirmary—To Close or Not to Close—War Nomenclature of Wards—War's Effect on Women—Manchester Doctors and the War—Poor-Law Medical Officers in War Hospitals—Payment for Medical Attendance on Soldiers—A Medical Superintendent's Honorarium—Medical Staff Changes under the M.A.B.—Women Dispensers—Windfalls for Brighton Hospitals—Hospitals and the Mineral-Water Tax—Insurance Benefits and Salaries—This Week's Drug Market.

**Who is Insane?** ... ... ... ... ... ... 49

By Charles Arthur Mercier, M.D.Lond., F.R.C.P.

**Typhoid Fever** ... ... ... ... ... ... 50

Treatment in the Army.

**From Across the Seas** ... ... ... ... ... 51

Meat Prices; Cooking; The Staff's Liabilities.

**Parliament and the Profession** ... ... ... 52

**The Matrons' and Sisters' Department** ... 53

The Glad Eye of Observation.
Round the Hospitals.
Nurses' Registers—Matrons as Nurse-Trainers—Pensions for War Nurses—Butter Substitutes—Matrons' Worries—A Wonderful Memory.

**Textile Supplies for Hospitals** ... ... ... 55

IX.—Cotton Uniforms.

**Criminal Germans and British Prisoners** ... 57

A Terrible Story of Brutal Cruelty: Heroic British Practitioners.

PAGE

**Hospital Results in 1915** ... ... ... ... ... 59

More Early Reports of the Voluntary Hospitals.

**Passing Events and Topics** ... ... ... ... 60

**The Editor's Letter-Box** ... ... ... ... 61

Some Difficulties with V.A.D. Workers—Vacancies for Midwives—Tubercular Patients on a Hill—The Royal Hospital for Incurables—"Scotland's First Open-Air Hospital."

**Gifts to Hospitals** ... ... ... ... ... ... 62

**Personalia** ... ... ... ... ... ... ... 62

**Obituary** ... ... ... ... ... ... ... 62

**Matrons' and Sisters' Appointments** ... ... 62

**The College of Nursing: Great Meeting at St. Thomas's Hospital: Full Verbatim Report** ... ... ... ... ... ... ... 63

The Audience — How the College of Nursing Originated—Hospitals and Nurse-Training Schools Approached—Three Fundamental Principles Agreed to—The Foundation and Establishment of the College—The Constitution of the College—The Whole Nursing Profession Represented in the Management—Working Committees: Their Formation and Duties—The Work of the College to be Educational—State Recognition or Registration—The Present is the Moment to go Ahead—Very Many Large Training Schools Support the College—THE DISCUSSION—The Representation of Ireland—An Amusing Speech—How Council Elected in 1918—The V.A.D.s and the College—Questions for the Consultative Board to Determine—Some Questions for the Consultative Board—Some Passing Matters—Professor Ritchie of Edinburgh—The Memorandum of Association—Some Published Registers of Nurses—The Royal British Nurses' Association—A Matron's Confidential Register of Nurses—The Next Meeting of Hospital Representatives—Lancashire Guardians and the College of Nursing—Lord Sandhurst Gives Sympathetic Support—A Very Long Way to Success.

**Accuracy of Medicine Measures** ... ... ... vi

